# Toe spreading ability in men with chronic pelvic pain syndrome

**DOI:** 10.1186/1471-2490-5-11

**Published:** 2005-06-10

**Authors:** Ugur Yilmaz, Ivan Rothman, Marcia A Ciol, Claire C Yang, Richard E Berger

**Affiliations:** 1 Department of Urology, University of Washington, Seattle, Washington, USA; 2 Department of Urology, University of Washington, Seattle, Washington, USA; 3 Rehabilitation Medicine, University of Washington, Seattle, Washington, USA

## Abstract

**Background:**

We examined toe-spreading ability in subjects with chronic pelvic pain syndrome (CPPS) to test the hypothesis that subjects with CPPS could have deficiencies in lower extremity functions innervated by sacral spinal roots.

**Methods:**

Seventy two subjects with CPPS and 98 volunteer controls were examined as part of a larger study on CPPS. All the subjects underwent a detailed urologic and neurological examination including a toe-spreading examination with a quantitative scoring system. We compared the groups in terms of ability of toe-spreading as either "complete" (all toes spreading) or "incomplete" (at least one interdigital space not spreading) and also by comparing the number of interdigital spaces. For CPPS subjects only, we also analyzed the variation of the National Institutes of Health Chronic Prostatitis Symptom Index (NIH-CPSI) scales by toe-spreading categories.

**Results:**

CPPS subjects were less often able to spread all toes than subjects without CPPS (p = 0.005). None of the NIH-CPSI sub-scales (pain, urinary symptoms, and quality of life), nor the total score showed an association with toe spreading ability.

**Conclusion:**

We found toe spreading to be diminished in subjects with CPPS. We hypothesize that incomplete toe spreading in subjects with CPPS may be related to subtle deficits involving the most caudal part of the spinal segments.

## Background

Male chronic pelvic pain syndrome (CPPS Type III) is a new term for disease entities previously known as chronic abacterial prostatitis (CPPS Type IIIa) and prostatodynia (CPPS Type IIIb) [1]. The syndrome is characterized by chronic pain of the perineum, lower back, inguinal, scrotal and suprapubic region without findings of infection [2,3]. Male CPPS is associated with approximately 2 million office visits per year in the U.S. and decreases the quality of life of those affected to a similar extent as major depression, myocardial infarction, angina, or Crohn's disease [4-7]. Many chronic prostatitis studies have focused on microbiology and prostatic inflammation [2,8,9]. Few studies have examined subjects with CPPS neurologically [10-12]. We compared neurological exams, including S2-S3 function as reflected in toe spreading, in subjects with and without CPPS.

The lumbosacral spinal cord innervates the pelvis and lower extremities and it is a cross-road for both the afferent nerves related to CPPS and the nerves innervating the lower extremities. The tibial nerve is a branch of the sciatic nerve and is formed by spinal segments from L4 to S3 roots. Toe-spreading is a reflection of tibial nerve function, and neural innervation for this function is derived from the S2 and S3 roots, the roots that innervate much of the pelvis [13]. In previous studies we have found pelvic muscular abnormalities in subjects with CPPS [14]. Therefore, we examined the hypothesis that subjects with CPPS would have more difficulty spreading their toes than subjects without CPPS. If we found this to be so, it could be a reflection of abnormalities in the central or peripheral co-ordination of pelvic function, which could be etiologically related to CPPS.

## Methods

Seventy two subjects with CPPS and 98 subjects without CPPS were examined as part of a larger study on CPPS. Subjects with CPPS were recruited from the prostatitis clinic at the University of Washington and control subjects without pelvic pain via advertisements on bulletin boards and local newspapers. Human Subjects Review Committee approval and written informed consents from both the subjects with CPPS and controls were obtained. All subjects provided urologic and neurological history. Inclusion criteria for pain subjects included symptoms of pain in the pelvis lasting at least for 3 months including the perineum, lower back, abdomen, rectum, testicles, or penis, with or without pain during urination or ejaculation. Exclusion criteria for all subjects included current urinary tract infection; post-surgical pain, previous radiation therapy, treatment for bladder, prostate, renal or other urinary malignancies; history of genitourinary tuberculosis; any known neurological abnormalities including spinal cord injury; overt psychiatric disease; and subject's age under 18 or over 65. The controls had no pelvic pain and were not on pain treatment of any sort.

All subjects with CPPS completed a National Institutes of Health Chronic Prostatitis Symptom Index (NIH-CPSI) [15]. The symptoms were scored on three domains: pain, urinary symptom and quality of life. All subjects underwent a detailed urologic and neurological examination including a toe-spreading examination with a quantitative scoring system which consisted of scores from 0 (no spreading) to 4 (spreading of all toes) as measured from the number of interdigital spaces the subjects could produce when asked to spread/fan their toes. For instance, the score was 3 if only three of interdigital spaces could be opened. In this study no record was made of the location of the interdigital spaces.

Toe-spreading scores were compared between pain subjects and healthy volunteers using a permutation test based on the method developed by Berger et al[16], using a program written in the R language (The R Foundation for Statistical Computing, Version 1.8.1, 2003). Chi-square tests were performed to compare categorical variables between the control group and subjects with CPPS, while t-tests were used for continuous variables, using a significance level of 0.05. Spearman correlation was performed to assess association between NIH scales, age, and toe spreading ability among pain subjects.

## Results

Twelve CPPS subjects were excluded from the analysis due to missing values, since the toe spreading exam was not instituted as part of the study until after these subjects had enrolled. Therefore, the analyses were based on the remaining 60 CPPS subjects. The mean (standard deviation) ages of the 60 subjects and 98 controls were 40.4(10.4) and 34.2(10.4) years, respectively (p < 0.001). The two groups differed also in employment and educational level (p = 0.021 and p < 0.001, respectively), but not in race distribution and marital status (p = 0.547 and 0.123, respectively). The latter differences might be attributed to the age difference between groups (younger people are less likely to be graduated from higher education or employed fulltime, for example).

Table 1 shows the distribution of interdigital spaces on each foot by group (CPPS or control subject). Of the 60 CPPS subjects, 3 (5%) were able to spread all toes on both feet while 22 (22.5%) of the 98 controls were able to spread all toes (p = 0.007). Toe-spreading on the right side was more likely to be complete in the controls than the CPPS subjects (p = 0.032). The ability of toe spreading was similar in both groups on the left side (p = 0.129).

**Table 1:** Number of interdigitial spaces by foot and group*

Right foot scores		Left foot scores				
		
		0	1	2	3	4
Controls n = 98	0	**9**	1	1		
	1	5	**17**	1	2	1
	2	1	3	**14**	3	
	3			1	**9**	
	4			1	7	**22**

Pain subjects n = 60	0	**10**	3			
	1	3	**9**	6		
	2			**9**	3	2
	3	1	1	1	**5**	1
	4	1			2	**3**

*The bold-typed numbers represent the number of subjects with similar toe-spreading ability on both sides, as defined by the number of interdigital spaces. For example, 17 controls could display one interdigital space in each foot, while nine of the pain subjects were able to do so.

To test for differences between the two groups, we also considered the total number of interdigitial spaces from both feet as the outcome of interest. This outcome can be ordered from 0 to 8, and two people with the same number of interdigital spaces were considered to be "equal". Thus, a person with total number of spaces of 4 could have 3 spaces in one foot and 1 in the other, or 2 spaces in each foot. When comparing a control subject with a CPPS subject, we say that the CPPS is "favored" if he has a larger number of spaces than that of the control subject. Pairs where both subjects have the same number of interdigital space are excluded from the analysis, since they do not contribute to differentiating the two groups. The null hypothesis is: the proportion of times that CPPS is "favored" among all comparisons between CPPS and control subjects is 0.5 (half of the time), against the alternative that the proportion is less than 0.5. Using the data from Table 1, we observed 1909 comparisons where the CPPS subjects was "favored" among 5202 comparisons (proportion = 0.367). A permutation test using Monte Carlo simulation (run 10,000 times) yielded a p-value of 0.005.

The association between toe spreading ability (for each foot separately and combined), age, marital status, education and NIH scales was assessed using visual displays and Spearman correlation. The correlations (not shown here) were of very small magnitude and none were statistically significant.

## Discussion

In the present observational and descriptive study, we found toe spreading to be diminished in subjects with CPPS. Subjects with CPPS were less able to spread their toes than subjects without CPPS. This neurological finding could be a consequence of sacral nerve processes resulting in other neurologically related findings in CPPS such as bladder hypersensitivity, pelvic floor spasm and tenderness, lower urinary flow rates, and perineal heat hypersensitivity [11,12,17]. Our findings suggest that muscles innervated by the sacral nervous system are often in spasm and hypersensitive in subjects with CPPS [14].

Toe-spreading is a function of spinal segments S2 (flexion/extension) and S3 (abduction) and, neurological functions involving intrinsic muscles of the feet and toes reflect the neural integrity of the sacral segments. Thus, foot intrinsic muscles share the same innervation as the muscles of the pelvic floor. If there is no other cause for motor deficits in the lower extremity, such as peripheral nerve injury, the patient with motor deficits in the feet will have corresponding deficits in pelvic floor innervation. The sural nerve originates from the union of the medial sural cutaneous branch of the tibial nerve and the sural communicating branch of the common peroneal nerve. Treating certain bladder dysfunctions by afferent nerve neuromodulation techniques such as sural nerve stimulation is another example of neural intimacy and the central association between lower extremity and pelvic visceral functions [18]. Percutaneous tibial nerve stimulation has also been successfully used in the neuromodulative treatment of CPPS as well as overactive bladder [19,20].

Women with CPPS have an increased incidence of facet joint degeneration secondary to intervertebral disc degeneration [21]. In some cases this causes referred pain to the pelvic region, which suggests that further studies of spinal column abnormalities and CPPS are needed. There is great variation in the interossei muscles of foot and the attachments can vary widely and contribute to stabilization of the forefoot when the heel is off the ground. Mild foot dysfunction as in incomplete toe-spreading might also be related to subtle congenital deficits involving the most caudal part of the spinal segments and thereby affecting simultaneously both the pelvis and lower extremities. This hypothesis is supported by the occurrence of defects during embryogenesis involving the caudal part of the neural tube [22].

Another explanation for our findings might be muscle dysfunction related to nerve entrapment or injury that might affect both the foot and the pelvis. Pudendal nerve entrapment may cause neuropathic pain related to CPPS, resulting from aberrant development and subsequent malpositioning of the ischial spine associated with athletic activities or during the period of development and ossification of the spinous process of the ischium [23]. Pudendal canal syndrome may be a somatic cause for CPPS; however, further studies are needed to delineate the exact relation of pudendal nerve entrapment to CPPS. In a study involving posttraumatic piriformis syndrome, surgical release of adhesions between the piriformis muscle, the sciatic nerve, and the roof of the greater sciatic notch remarkably relieved lower extremity findings [24].

The genetic features of CPPS have not been extensively studied but it may sometimes cluster with interstitial cystitis [25]. There are familial and sporadic cases with neuropathy associated with genetic abnormalities such as 17p11.2 deletion, forming hereditary neuropathy associated with liability to pressure palsies, and electrophysiologic examinations have shown the presence of conduction abnormalities mostly located at the common entrapment sites [26,27]. Although rarely encountered, the hereditary liability to nerve entrapment syndromes could be among the underlying causes associated with CPPS and should be examined.

We also found incomplete toe spreading in a substantial number of healthy control subjects. This suggests that incomplete toe spreading may not be sufficient by itself to cause CPPS or that our controls may be at increased risk to develop CPPS later in life. It is possible to find functionally or morphologically abnormal findings in healthy control subjects as well as in cases with sacroiliac joint abnormalities or low back pain [28,29]. In our opinion, a primary etiologic factor leading to incomplete toe spreading in subjects with CPPS could be either a subtle neural deficit for lower extremity function or a spinal cord abnormality leading to both pelvic pain and incomplete toe spreading. Either could theoretically be related to the development of pelvic muscular pain.

We did not analyze all the combinations of toe spreading because the specific toes between which spreading occurred were not recorded. Furthermore, we did not have data on dominant side and future studies should collect that information. The age difference between the two groups can pose a limitation to the study since aging could also result in subtle changes in toe spreading ability, however, we did not find a relationship between age and toe spreading ability in our sample. Our present observational study provides impetus to study neurological phenomenon on CPPS subjects.

## Conclusion

We found that subjects with CPPS were less able to spread their toes than subjects without CPPS in an observational study. This finding could be related to sacral abnormalities which might be involved in the etiology of CPPS. Our study protocol did not involve extensive neurological and neurophysiologic evaluations. Future studies on spinal bone and ligamentous integrity, podiatry and neurological abnormalities are warranted.

## List of abbreviations

CPPS: Chronic pelvic pain syndrome

NIH-CPSI: National Institutes of Health Chronic Prostatitis Symptom Index

## Competing interests

The author(s) declare that they have no competing interests.

## Authors' contributions

UY participated in the data evaluation, analyses and presentation of the study. IR participated in the design and data collection. MAC participated in the design of the study and statistical analysis. CCY participated in the presentation of the study. REB participated in the design, data collection, evaluation, analyses and the presentation of the study.

**Figure 1:**
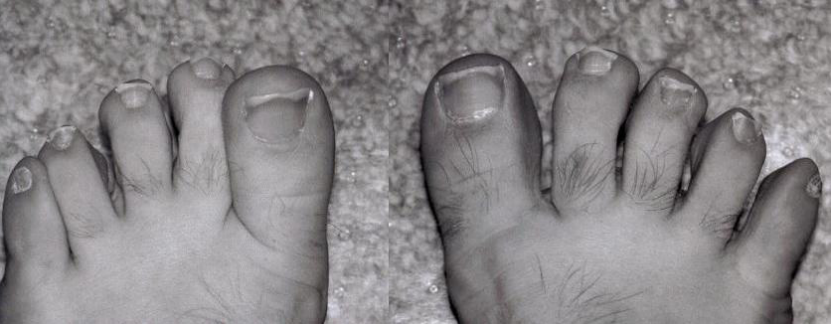
Toe-spreading is demonstrated. On the left is a score 2, on the right a score 4.

## Pre-publication history

The pre-publication history for this paper can be accessed here:


